# Fasciitis Necroticans after Elective Hernia Inguinal Surgery

**DOI:** 10.1155/2014/981262

**Published:** 2014-01-05

**Authors:** T. A. Sigterman, Kim J. Gorissen, Dennis E. J. G. J. Dolmans

**Affiliations:** ^1^Atrium Medical Centre, Department of General Surgery, Henri Dunantstraat 5, 6419 PC Heerlen, The Netherlands; ^2^Oxford University Hospitals, Department of Surgery, Old Road, Headington, Oxford OX3 7LE, UK; ^3^Diakonessenhuis, Department of Surgery, Bosboomstraat 3, 3582 KE Utrecht, The Netherlands

## Abstract

Necrotising fasciitis is a rare but disastrous complication after elective surgery. We present two patients (both male, 58 and 18 years old) who developed necrotising fasciitis following elective inguinal hernia repair according to Lichtenstein. The importance of both recognition and time interval between symptom occurrence and surgical intervention is illustrated, emphasising the need for immediate action when necrotising fasciitis is suspected. A high index of suspicion of necrotising fasciitis should be maintained when a wound infection is accompanied by disproportional pain, lethargy, or sepsis. Epidermolysis and subcutaneous emphysema are often very late symptoms. Recognition and immediate intervention decrease mortality and morbidity.

## 1. Case Report 

**Figure 1:**
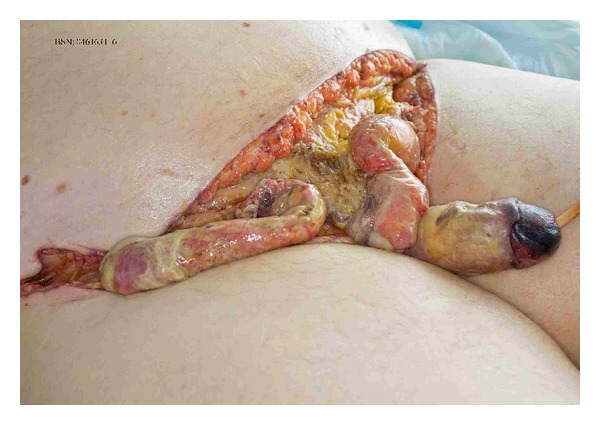


A 58-year-old man undergoes an elective correction of an inguinal hernia according to Lichtenstein [1]. His medical history includes insulin dependent diabetes and hypertension. The procedure was straightforward and the patient was discharged the same day. Two days after the operation the patient visited our emergency room with severe pain and swelling at the operation site. On physical examination we saw a moderately ill man, with a temperature of 38.7 degrees Celsius and a pulse rate of 101 beats per minute. Blood examination showed leukocyte numbers of 9.9 × 109 and a CRP 179 mg/L. The surgical site showed a hematoma without redness or pus. The patient was admitted and reassessed after eight hours. Moreover an ill man was seen with blistering, livid discoloration of the scrotum. With the suspicion of a fasciitis necroticans (FN) the patient was brought to the operation theatre and antibiotics were started. Perioperatively a fulminant Fournier gangrene [2] was seen, for which an extensive necrosectomy was performed with the formation of a colostomy (Figure 1). Perioperative cultures showed *group A beta-hemolytic streptococcus* (GAS). The patient was admitted to the intensive care unit (ICU). After a total of nine reinterventions and two months in hospital, he was discharged to a rehabilitation centre.

**Figure 2:**
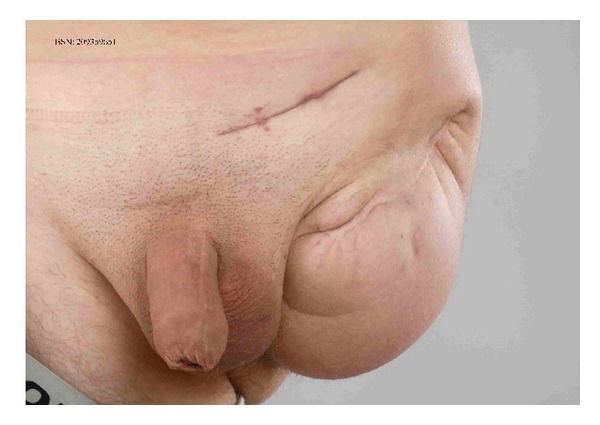


Secondly, an 18-year-old male underwent elective inguinal hernia surgery according to Lichtenstein. Medical history showed a through-the-hip amputation in its first weeks of life because of iatrogenic dissection of the femoral artery. The surgical procedure was straightforward, whereafter the patient was discharged the same day. That evening he felt unwell; after a few hours he visited the emergency room with groin pain and fever. The patient was severely ill with a temperature of 39.1 degrees Celsius and a pulse rate of 92 beats per minute. Blood tests showed leukocyte numbers of 23 × 109 and a CRP of 365 mg/L. There was redness of the wound, which was painful on palpation, without blisters or crepitation. On exploration of the wound in the operation theatre, a large amount of foul smelling fluid was drained. Blood and wound cultures showed a GAS. After three days in the ICU the patient was transferred to the surgical ward. The patient was also treated for one week intravenously and two weeks orally with amoxicillin/clavulanic acid and clindamycin. The patient recovered well without any sequelae and is discharged in good clinical condition (Figure 2).

## 2. Introduction

We describe the case histories of two patients following elective inguinal hernia correction complicated by fasciitis necroticans, to focus attention on rapid recognition of this potentially lethal complication. The occurrence of FN after surgery is rare. To recognize and differentiate, by specialists and general practitioners, between FN and a simple wound infection in combination with direct appropriate action is vital. General practitioners, nursing, and emergency physicians should recognize this condition easily, because more operations such as inguinal hernia surgery are carried out in day care.

## 3. Discussion 

FN is a bacterial infection of the subcutaneous tissue that spreads across the fascia [3]. FN can occur all over the body, where infection around the perineum is called Fournier gangrene [2]. Less than 50% of cases have an identifiable cause of infection, where it often concerns a minimal Porte d'entree point, like an insect bite, a scrape, or a cut [3]. The occurrence of FN after surgery is rare, described in less than 0.5% [4]. A few case reports of FN after elective hernia inguinal repair are described, of which three are after Lichtenstein [5, 6] and one after totally extraperitoneal approach [7]. The two patients presented in one year after another. In those two years 628 patients underwent an inguinal hernia repair according to Lichtenstein. Both patients have been operated by different surgeons and also by different assistants. The operation room and surgical instruments have been checked and there was no causal relation between personnel, operation room, and instruments and the FN. No prophylactic antibiotic therapy was given according to local protocol.

Etiologically two subtypes of FN can be distinguished [2, 8]. Type 1 results from a polymicrobial infection, in which an average of 4 different organisms is found, often gram-positive cocci, gram-negative rods, and anaerobes [2]. This type occurs in 55–75% of cases [8]. It is more often seen in immunocompromised patients. Polymicrobial FN can also be seen at low gastrointestinal perforations, perforated strangulated herniations, perforated diverticulitis, or colorectal carcinomas. Coinfection with *Clostridium perfringens* leads to gas gangrene [3]. Type 2 FN is caused by GAS, *Streptococcus pyogenes*, sometimes in combination with *Staphylococcus aureus*/MRSA. This subtype is manifested especially in the young, healthy, immunocompetent hosts [9]. GAS produces several exotoxins including streptolysin O, exotoxins A, B, and C, M1 and M3 surface antigens, and super antigens. As a result, overstimulated macrophages release large amounts of TNF-alpha, IL-1, and IL-6, which lead to the systemic inflammatory response syndrome (SIRS), sepsis, multiple organ failure, and/or death [10]. The prevalence of asymptomatic carriage of GAS is 15–20% in children and 2.1% in adults [9]. Annually, some 320 cases of FN are reported in The Netherlands.

FN is often accompanied with disproportionate pain in the infected area. Rapidly expanding redness of skin, extending in up to three centimetres per hour, can be seen [3, 8]. Through thrombosis of vasculature of the skin, necrosis of subcutaneous fat and skin takes place, on which blue/purple discolorations occur, eventually leading to blistering. In the case of gas formation crepitations can be felt, but this is seen in less than 30% of the cases [3]. Patients are generally ill, have high fever, and can suffer from vomiting and diarrhoea. Septic shock occurs within 24 to 48 hours. Despite advances in treatment and support, FN still has a mortality of 25–35% [4]. Serum lactate is known as a good predictor of sepsis severity and mortality. The infected tissues drain typically foul smelling moisture, often described as dishwater. A positive wound and/or blood culture with GAS confirmed the diagnosis eventually.

Suspicion of FN must lead to acute intervention: surgical debridement, antibiotics, and hemodynamic support during sepsis.

Surgical debridement should be performed as soon and completely as possible. Mortality rates increase up to ninefold within the first 24 hours when no or inadequate debridements are done [11]. Wide excision in any case beyond redness of the skin into highly vascularized subcutis and vital fascia is essential. On average, three operations are needed with intervals between 12 hours and 36 hours to obtain control. In FN of the extremities sometimes amputation of a limb must be considered to prevent spread [4]. In Fournier gangrene a diverting colostomy may strongly improve wound care of the perineum [7].

Antibiotics cannot penetrate into necrotic tissue. They serve as an adjunct to adequate surgical therapy. Recommendation of the Working Party on Antibiotic Policy Foundation is shown in Table 1. Clindamycin covers anaerobes and inhibits the M protein and exotoxin synthesis of the GAS. The average duration of antibiotic treatment is 10–14 days.

## 4. Conclusion

Fasciitis necroticans is a rare but serious complication after (inguinal hernia) surgery. The key is to differentiate it from a regular wound infection at the very early occurrence after surgery with rapid progression. Disproportionate pain, malaise, or sepsis needs to raise suspicion. Blisters and crepitations are pathognomonic for the disease but often occur in a (too) late phase. Atypical variants are common. The cases presented show the importance of early recognition of FN and acute and aggressive surgical intervention and antibiotic treatment. Because the majority of inguinal hernia repair is performed in day care surgery it is important that primary care physicians and emergency room emergency physicians are aware of this serious complication.

## Figures and Tables

**Table 1:** Foundation working party on antibiotic policy necrotizing fasciitis.

Type	Advice	
Unknown pathogen	(i) Penicillin 2 Million E	6 dd iv +
	(ii) clindamycin 600 mg	3 dd iv +
	(iii) with aminoglycoside	1 dd iv
	or	
	(iv) clindamycin 600 mg	3 dd iv +
	(v) aminoglycoside	1 dd iv
	or	
	(vi) penicillin 2 milj E	6 dd iv +
	(vii) clindamycin 600 mg	3 dd iv +
	(viii) ciprofloxacin 400 mg	2-3 dd iv

Type 1: aerobe and anaerobe mixed flora	(i) Amoxicillin 1000 mg	4 dd iv
	(ii) aminoglycoside	1 dd iv +
	(iii) metronidazole 500 mg	3 dd iv
	or	
	(iv) amoxicillin-clavulanic acid 1000/200 mg	6 dd
	(v) clindamycin 600 mg	3 dd iv of oral
	(vi) with aminoglycoside	
	or	
	(vii) cefuroxime 750–1500 mg	3 dd iv
	(viii) metronidazole 500 mg	3 dd iv +
	(ix) aminoglycoside	1 dd iv once

Type 2: GAS	(i) Penicillin 1 tot 2 milj E	6 dd iv +
	(ii) clindamycin 600 mg	3 dd iv
	or in allergy for beta-lactam:	
	(a) cefuroxime 750–1500 mg	3 dd
	(b) or cefotaxime 1000 mg	4 dd
	(c) or ceftriaxone 2000 mg	1 dd iv +
	(ii) clindamycin 600 mg	3 dd iv

GAS: Group A beta-haemolytic streptococ; dd: de die, per day; iv: intravenous.

## Abbreviations

FN: Fasciitis necroticans
GAS: *Group A beta-haemolytic streptococcus*.
